# Notes on Practical Surgery

**Published:** 1886-06

**Authors:** W. J. Penny

**Affiliations:** Clifton


### Periscope of Medical and Surgical Progress.

---

### NOTES ON PRACTICAL SURGERY.

By W. J. Penny, F.R.C.S., Clifton.

Ulceration of the Trachea and Innominate Artery after Tracheotomy.—Mr. R. W. Pughe records the case of a child two years old, on whom tracheotomy was performed as a preliminary to laryngotomy, for the removal of warts from the larynx. The tube used was Fuller's, with a split or dilating outer canula. Bronchitis supervened; but after a few days the child was apparently doing well. On the seventh day after the operation severe arterial hæmorrhage occurred from the trachea, with fatal result. At the autopsy, the trachea and innominate artery opposite the lower end of the tracheotomy tube were found to be ulcerated. Mr. Pughe ascribes the ulceration to pressure, caused by too great curvature of the lower part of the tube. He recommends Mr. R. W. Parker's tube, which has the lower part straight and both canulæ movable on the outside shield.—*Liverpool Medico-Chirurgical Journal*, January, 1886.

Epicystotomy.—Sir Henry Thompson, in his book *The Supra-pubic Operation of Opening the Bladder*, recommends this operation for the removal of some tumours and of calculi too large or too hard to be removed by lithotrity. He states his belief, that in the hands of most operating surgeons this proceeding will prove safer and easier than lithotrity, for hard stones over 1½ oz. in weight. Sir Henry describes the special dangers of epicystotomy to be wound of the peritoneum and urinary infiltration; those of the lateral operation, wound of the rectum, hæmorrhage, bruising and splitting of the tissues round the neck of the bladder. He advocates the use of Petersen's bag in the rectum, injects the bladder with from six to ten ounces of boracic lotion, but contrary to the practice of Sir Joseph Lister, does not sew up the wound in the bladder after extracting the stone or tumour. In some cases Sir Henry inserts a drainage tube into the bladder above the pubes. The wound is allowed to heal by granulation.

CASE OF AMPUTATION AT THE HIP-JOINT, IN WHICH RE-INJECTION OF BLOOD WAS PERFORMED, AND RAPID RECOVERY TOOK PLACE.—The patient, a boy of eighteen, very emaciated and anæmic, with strumous disease of both hips, left knee and left elbow, was operated on by A. G. Miller, M.D., in the Royal Infirmary, Edinburgh. Dr. Duncan collected the greater part of the blood which escaped from the limb in a vessel containing a solution of phosphate of soda, and after the arteries had been tied, injected the mixture into the deep femoral vein. The patient did not suffer from shock after the operation, nor had he any depression of temperature. Healing rapidly took place. Dr. Miller considers it unlikely that the boy would have survived had not the greater part of the blood been re-injected.—*Edinburgh Medical Journal*, Feoruary, 1886.

Dr. Duncan reports some similar cases in the *British Medical Journal* of January 30th, 1886. He uses a 5 p.c. solution of phosphate of soda, and mixes this with three times as much blood. The phosphate of soda delays coagulation.

NOTES ON THE TREATMENT OF GONORRHŒA.—Surgeon E. J. Erskine Risk reports forty-eight cases. Thirty-one treated from the initial and acute stage by injection of potassæ permanganas, one grain to one ounce of water, had a duration of 15.74 days. Seventeen cases treated at first with warm water injections, and when the acute stage was over by injections of chloride of zinc, half a grain to the ounce, averaged 24.1 days.—*Indian Medical Journal*, Jan., 1886.

Surgeon R. H. Firth (*Indian Medical Journal*, April, 1886), publishes the results of 413 cases. Forty-nine treated with warm corrosive sublimate injections, one-sixteenth of a grain to the ounce, averaged 17.5 days; fifty-one treated with the same lotion cold, averaged 20·1 days. Thirty treated with permanganate of potash injections, one grain to one ounce, averaged 19.9 days. All the above cases were treated with antiseptic injections, on the theory of destroying the gonococcus or specific germ of gonorrhœa. Surgeon Firth lays great stress on the importance of using warm solutions.

Diagnosis of Gonorrhœa in the Female. — Dr. Martineau claims that a simple may be distinguished from a specific vaginal discharge by the simple expedient of using a piece of litmus paper. In the specific form the discharge is always acid, while in the simple it is always alkaline.—*American Practitioner and News*, February 20th, 1886.

Cocaine Anæsthesia in Major Surgical Operations.—*Femoral Supra-condyloid Osteotomy in a Boy aged four.*—Numerous injections of a 5 p.c. solution of cocaine were used. The second and subsequent injections were made into the periphery of the area of redness caused by the preceding. No pain was experienced by the boy, except at the first puncture of the needle and during the straightening and fixing of the leg to the splint. Three grains of cocaine were used in this operation.

*Excision of the Hip-Joint in a Girl of six.*—The superficial and deep structures were cocainised in the same manner as in the previous case; but owing to the supply of cocaine running short, the anæsthesia was not so profound.—Milton Josiah Roberts, M.D., *New York Medical Journal*, October, 1885.

*Amputation of the Thigh in a Man of Sixty-nine.*—The patient, a man of intemperate habits but otherwise healthy, sustained a compound fracture of the leg. An attempt was made to save the limb; but after five weeks, amputation was decided on. The administration of ether was attended with such alarming symptoms that it was abandoned. One week after, it was decided to try the effect of cocaine by Dr. J. L. Corning's method. The operation by antero-posterior flaps was selected, cutting from without inwards for the anterior, then transfixing and making the posterior flap from within outwards. The patient complained of no pain except during the sawing of the bone and once when the knife was carried beyond the parts anæsthetised. The strength of the solutions of cocaine used was one per cent. for the skin, and one-half per cent. for the deeper tissues.—Theodore R. Varick, M.D., *New York Medical Journal*, February 20th, 1886.

Mr. A. E. Maylard (*Glasgow Medical Journal*, February, 1886) suggests that the application of a solution of cocaine to the surface of the wound immediately after the operation and before the dressing may be the means of mitigating much subsequent suffering.

Dr. J. L. Corning (*New York Medical Journal*, January 2nd, 1886) recommends the use of weak solutions of cocaine—one, one-half, or even one-third per cent.—injected in small quantities throughout the entire field of operation, superficial and deep. When a limb is to be operated on, he applies an Esmarch's bandage on the distal side of the site of operation, then makes the injections, and then fastens a tourniquet on the proximal side. If more is required during the operation, he re-applies the Esmarch, removes the tourniquet, makes the injection, and immediately re-applies the tourniquet. Anæsthesia has been maintained by these means for two hours at a time.

Permanent Drainage in Ascites.—Dr. Aug. G. Caillé relates two cases. The first patient was an elderly gentleman, suffering from cirrhosis of the liver. The symptoms depending on ascites were so marked as urgently to demand tapping and repetition of the operation. In the course of seven months he was tapped nine times, a pailfull of serum being removed on each occasion. When tapping was next required, Dr. Caillé inserted an india-rubber tube through the canula; this was left in for nine weeks; six weeks after its removal, the opening closed. After drainage had become established, the œdema disappeared rapidly, the heart's action improved, the breathing was freer, the troublesome cough disappeared, the bowels became regular, the appetite improved, and the patient was able to go about wearing the tube. For nine months he resumed his duties, there being no return of œdema or ascites. After that time his strength began to fail, and he finally died of heart-failure. Two weeks before death, the ankles became slightly œdematous. At the autopsy, cirrhosis of the liver and fatty heart were found. Careful inspection of the peritoneum at the seat of puncture revealed no evidence of inflammation.

The second case, somewhat similar, was complicated with marked purpura hæmorrhagica. The tube was worn for seven weeks, great relief being experienced; and the patient enjoyed a comfortable existence for some months, ultimately dying of heart-failure. No autopsy was allowed.—*Philadelphia Medical Times*, Feb. 20th, 1886.

Contagious Infection in Surgery.—Keummell, of Hamburg, performed a series of experiments with the view of gaining some definite rules for the purification of hands, instruments, and sponges. His investigations were so conducted that the objects to be examined were brought into contact with the nutritive soil employed by Koch. If no development occurred, the disinfection was considered complete—highly-polished new instruments were not disinfected by immersion for two minutes in a 5 per cent. solution of carbolic acid; but ten minutes in the same solution sufficed to purify them. Submersion for fifteen minutes in 1–1000 sublimate solution did not disinfect. Instruments with uneven surfaces, and those which had already been used and cleaned in the ordinary manner, required longer immersion. Those used in autopsies were not pure after fifteen minutes in 5 per cent. carbolic, or 1–1000 sublimate solution. If the instruments, even those used in autopsies, were first washed with warm water and a potash (green) soap, and then immersed for one minute in 5 per cent. carbolic or 1–1000 sublimate solution, they were made pure. Sponges should be treated in a similar manner, but require rather more soaking in the antiseptic fluid. After an autopsy, Keummell washed his hands several times with warm water and ordinary soap; in a few days the scratches made on the gelatine swarmed with micro-organisms. After an autopsy on a case of puerperal peritonitis, he found that ordinary soap and water with 5 per cent. carbolic did not purify his hands; but if they were washed with green soap and warm water, and then with a 5 per cent. solution of carbolic acid, no development occurred in the gelatine.

The practical application of Keummell's experiments lies in this: more stress should be laid on the employment in first order of green soap, warm water, and a nail-brush; after that an antiseptic, preferably a 5 per cent. solution of carbolic acid.—*Medical Press of Western New York*, April, 1886.

Regeneration of Tendons.—MM. Fargin and Assaki have excised portions of the tendons of animals, and connected the ends with a bridge of catgut, under strict antiseptic precautions. On examination at periods of two and three months afterwards, they found the catgut replaced by a fibrous formation resembling tendon. They have also grafted on, instead of the catgut, portions of tendons removed from other animals of the same and different species. By these means the experimentalists observed that union by first intention took place, notwithstanding the difference of species and of organisation.—*London Medical Record*, January, 1886.

Reparation of the Extensor Tendons of the Thumb.—The patient, a man aged 35, cut the back of his hand with glass two months before presenting himself. After the accident he was unable to extend his thumb, which was flexed on the palm of his hand. The cicatrix extended across the wrist-joint at the upper part of the tabatiere anatomique. On making an incision, the distal ends of the divided long and short extensors of the thumb came into view; the proximal could not be seen. Dr. Schwartz then exposed the tendon of the ext. carpi radialis longior, split it about 1½ inch from its insertion into the second metacarpal bone, leaving one half attached to the bone, and the other half with a free lower end; to this he attached the distal ends of the extensors of the thumb by suture. A good result ensued, both flexion and extension of the thumb being well performed, the latter somewhat peculiarly, the thumb being first adducted and then extended.—*Revue de Chirurgie*, November, 1885.

Elongation of a Muscle by Means of Pedunculated Muscle-Flap.—Professor Scriba, of Tokio University, Japan, in a case of contracture of divided tendons after compound fracture of the arm, united several of them by suture; but found that the extensor digitorum communis muscle could not be united to its tendon on account of loss of substance of the latter. To elongate the muscle, he divided it in its upper part transversely half-way through, and splitting the muscle longitudinally for some distance down, turned the flap thus formed back, and united its proximal end to the free edge of the tendon. Union took place by first intention, and free motion of the fingers was made possible. The author is of opinion that such plastic operations could not be done with healthy muscles; in this case the muscle was hard and shrunken.—*Annals of Surgery*, April, 1886.

Operation to Correct Deformity Resulting from Extensive Loss of Skin of the Arm.—The patient desired amputation on account of the loss of use, and deformity, resulting from cicatricial contraction after phlegmonous erysipelas of the arm. Mr. Bell, instead of amputating, removed three inches of the lower part of the humerus. The patient has now a useful arm with all the elbow movements.—*Edinburgh Medical Journal*, September, 1885.

The Radical Cure of Hydrocele.—Dr. William T. Bull comments on the various methods of radical cure of hydrocele. He states that the injection of iodine has no deaths at its door, but a large proportion of recurrences—44 in 523 cases, *i.e.*, 8.4 per cent. Acute suppuration supervened in five instances, or .95 per cent. The carbolic acid injection suggested by Levis, of Philadelphia, has as yet caused no fatal result. In 82 cases reported by Weir and Abbé, suppuration occurred in three, and one recurrence is noted. Dr. Keyes, in *The Medical Record*, February 20th, 1886, reports 50 cases treated by the carbolic acid injection with no accidents, and with perfectly satisfactory results. Dr. Bull mentions 343 cases of Volkman's operation (free antisepic incision with suture of the edges of the tunica vaginalis to the skin, and drainage), with three deaths, two from pyæmia and one from mercurial poisoning: a mortality of .83 per cent. Recurrence took place in five, or 1.45 per cent. Twenty-two cases of Bergman's modification of Volkman's operation are also quoted, with no deaths and no recurrences. Bergman extirpates all the tunica vaginalis except that covering the testicle. Dr. Bull, in summing up, states that in future he will first attempt cure by the injection of carbolic acid, unless further experience shows it to be less satisfactory than at present. In case of failure by this, and provided strict antiseptic precautions can be carried out, he will adopt "the only truly radical operation,"—that of extirpation of the tunica vaginalis. —*New York Medical Journal*, March 13th, 1886.

Dr. Keyes, after evacuating the hydrocele fluid, injects from thirty to sixty minims of pure carbolic acid deliquesced with glycerine.

Rare Case of Intestinal Malformation.—A male child, born in May, 1884, was found to have an imperforate anus. Dr. Jernegan dissected upwards in the ischio-rectal fossa; but failing to find the bowel, desisted and closed the wound. The next day the child began to pass fæces with the urine, every such passage giving pain. At the age of five months, symptoms of obstruction of the bowels set in; for five weeks nothing but urine was passed by the urethra, and for several days all nourishment was vomited. These symptoms passed away with the return of copious fæcal discharges with the urine. The child died in August, 1885, from the prostration induced by the hot weather and teething. At the *post-mortem*, the intestinal canal was normal, except at its lower part. There was entire absence of the rectum. The descending colon, with its sigmoid flexure dilated, distended, and forced to the right side, like the letter V, opened by a rigid tube, an inch and a half in length by half an inch in diameter, into the prostatic portion of the urethra. The bladder, which was not at all dilated, and to all appearances healthy, contained a quantity of liquid fæces. The urethra was injected, swollen, and dilated to such an extent that a large-sized catheter could easily be passed into the bladder.

Nothing abnormal was detected in the other abdominal organs.

Such cases as the above have a physiological as well as a pathological interest, and seem to be a reversion to a primitive type of vertebrate life.—Dr. E. P. HURD, *Boston Medical and Surgical Journal*, 1885.

THE TREATMENT OF FRACTURE OF THE PATELLA.—Professor Denis contributes an interesting and instructive paper on this subject, which would well repay perusal. During the last five years he has treated and observed, in three large metropolitan hospitals, sixty cases of fracture of the patella. Almost every variety of apparatus and treatment, except wiring, was used in these cases. In a few only has the union been osseous and the perfect function of the joint restored. In the great majority the results have been unsatisfactory, *i.e.*, when contrasted with the results after the wire suture. In many cases without wiring, the bond of union has been narrow, and the joint a fairly useful one.

Malgaigne states that he has never seen the function of the limb completely restored, even when the separation was limited to one-third of an inch. On the other hand, Professor Volkman states that in no case has he ever found bony union fail when the suture was employed.

Professor Denis adduces three arguments in favour of the operation of wiring the patella: 1st. The absence of great danger to life and limb. 2nd: The superior results as regards function of limb and joint. 3rd. The greater rapidity of repair.

As regards the objection, that equally good results can be obtained by the older methods of treatment, the analysis of the cases affords us a convincing argument to disprove that statement.

Appended to his paper is a table of 182 cases. These Professor Denis subdivides into two sections: 1st. 49 operated on before 1883, with seven deaths. 2nd. 133 operated on since 1883, with four deaths. The fatal cases before 1883 include those operated on by Barton in 1834, Gunn in 1864, Cabot in 1865, and Langenbeck in 1878. Of the other deaths, one was due to Bright's disease and *delirium tremens;* one to carbolic acid poisoning; in another, a surgeon assisted at the operation without purifying his hands, although they were bathed in the carbolic spray. The mortality previous to 1883, Professor Denis ascribes to the fact that the operation was in its infancy. He goes on to state: "With the gigantic strides which modern surgery is making, it is safe to predict that in careful hands this operation will be devoid of every risk to life."

Of 182 cases of fracture of the patella, comprising 81 recent, 53 old, 31 undefined, 12 compound, and 5 others, 75 are described as having a good result, 35 a fair, and 24 a poor result. There were 11 deaths; in 31 the result is not known, and 6 are still under treatment. Among the fair and poor results are included 1 case of amputation, 11 of total, and 9 of partial ankylosis following suppuration; 7 other cases suppurated (in two the result was ultimately good); 3 cases of total and 8 of partial ankylosis, apparently without suppuration.

The conclusions to which Professor Denis comes are these:—1st. In compound fractures of the patella there is not the slightest question as to the propriety of wiring the fragments. 2nd. In recent and old fractures, with the full permission of the patient, and under the strictest antiseptic precautions, the operation, in the light of present statistics, is wholly justifiable. 3rd. In debilitated patients, and in those suffering from any organic disease, the operation should not be employed, and is in fact contra-indicated, as all other operations of expediency. 4th. It is not an operation which can be indiscriminately performed, and never by an ordinary practitioner with little surgical experience, and with little faith in the germ theory of inflammation. 5th. The success of this operation depends wholly on conscientiously carrying out the smallest detail in aseptic surgery; and the surgeon who is not imbued with the true spirit of antiseptic surgery, is guilty of a criminal act towards humanity if he attempts this operation. 6th. While the number of cases yet operated on is too limited to admit of deductions, by means of which a final settlement of this question can be made in the minds of surgeons, the future practice of the surgery in America, the birthplace of this operation, and the practice of other countries, will soon enable us to condemn it, as an unsafe and unjustifiable procedure; or else it will raise it to a pinnacle from which we can recognise one of the grandest triumphs of our art.—*New York Medical Journal*, April 3rd and 10th, 1886.

